# AMPK: a master energy regulator for gonadal function

**DOI:** 10.3389/fnins.2015.00235

**Published:** 2015-07-14

**Authors:** Michael J. Bertoldo, Melanie Faure, Joëlle Dupont, Pascal Froment

**Affiliations:** ^1^Discipline of Obstetrics and Gynaecology, School of Women's and Children's Health, University of New South WalesSydney, NSW, Australia; ^2^Unité de Physiologie de la Reproduction et des Comportements, Institut National de la Recherche Agronomique, UMR85Nouzilly, France

**Keywords:** AMPK, testis, ovary, germ cells, fertility

## Abstract

From *C. elegans* to mammals (including humans), nutrition and energy metabolism significantly influence reproduction. At the cellular level, some detectors of energy status indicate whether energy reserves are abundant (obesity), or poor (diet restriction). One of these detectors is AMPK (5′ AMP-activated protein kinase), a protein kinase activated by ATP deficiency but also by several natural substances such as polyphenols or synthetic molecules like metformin, used in the treatment of insulin resistance. AMPK is expressed in muscle and liver, but also in the ovary and testis. This review focuses on the main effects of AMPK identified in gonadal cells. We describe the role of AMPK in gonadal steroidogenesis, in proliferation and survival of somatic gonadal cells and in the maturation of oocytes or spermatozoa. We discuss also the role of AMPK in germ and somatic cell interactions within the cumulus-oocyte complex and in the blood testis barrier. Finally, the interface in the gonad between AMPK and modification of metabolism is reported and discussion about the role of AMPK on fertility, in regards to the treatment of infertility associated with insulin resistance (male obesity, polycystic ovary syndrome).

## Introduction

The 5′ AMP-activated protein kinase (AMPK) is a serine/threonine heterotrimeric kinase composed of one catalytic α-subunit bound with β- and γ-regulatory subunits. The genes encoding the three subunits of AMPK are highly conserved in eukaryotic species including vertebrates, invertebrates, plants, fungi, and protozoa (Hardie et al., 2003; Ghillebert et al., 2011). Activation of AMPK occurs with the phosphorylation of the α-subunit at Threonine 172. AMPK is sensitive to the AMP to ATP ratio and is activated by an increasing AMP concentration and by the upstream kinases including liver kinase B1 (LKB1) and calcium/calmodulin (CaM) kinase (CaMKK) (Woods et al., 2003; Hawley et al., 2005). It can also be dephosphorylated by phosphatases [Protein kinase phosphatase-1 and -2A (PP2A and PP2C)] (Lu et al., 2010; Joseph et al., 2015). AMPK is activated in pathophysiological situations (exercise, stress), by metabolic hormones (leptin, adiponectin, ghrelin) or pharmacological agents [5-aminoimidazole-4-carboxamide-1-β-D-riboside (AICAR), metformin and thiazolidinediones (TZD)] (Hardie, 2015). It regulates energy homeostasis by maintaining constant intracellular ATP concentrations by stimulation of catabolic pathways and inhibition of anabolic pathways (Hardie, 2015). Several studies have also shown that AMPK is expressed in gonads (Tosca et al., 2005; Dupont et al., 2012; Tartarin et al., 2012a) and could play a key role in the reproductive function in linking the gonadal axis with energy balance. More precisely, AMPK is present in ovarian (granulosa and theca cells, oocytes and corpora lutea) and testicular (Sertoli, leydig and germinal cells) cells in different species [oyster (Guévelou et al., 2013), *C. elegans* (Lee et al., 2008), bird (Tosca et al., 2006a; Nguyen et al., 2014), mammals: cow (Tosca et al., 2007a), pig (Mayes et al., 2007), rodents (Tosca et al., 2005; Downs et al., 2010; Tartarin et al., 2012a) and human (Pellatt et al., 2011)]. This kinase controls gonad steroidogenesis and germinal cell maturation but also cell proliferation and survival, polarity, formation, and maintenance of cellular junctional complexes, and cytoskeletal dynamics. In this review we report briefly some of the known functions of AMPK in the female and male gonad, and then we describe the potential role of this kinase in the interactions between metabolism and gonadal function. Most of the studies and conclusions are based on animal studies. However, we reported human studies (about granulosa and thecal cells or human embryonic testis) when it was possible.

## Gonadal steroidogenesis

In female mammals and birds, the role of AMPK has been studied in detail in granulosa cell cultures by using pharmacological agents and adenovirus-mediated expression of dominant negative forms of AMPK (Tosca et al., 2005). AMPK activators inhibit the secretion of progesterone and/or estradiol by granulosa cells in mammals (Tosca et al., 2005, 2007a). In rat and bovine species, this inhibition is associated with a decrease in 3beta-hydroxysteroid dehydrogenase (3β-HSD) mRNA and protein levels and a decrease in MAP kinase (MAPK) extracellular signal-regulated kinases (ERK) 1/2 phosphorylation (Tosca et al., 2005, 2007a, 2010). In rats, AMPK activation induced by metformin does not reduce aromatase expression and estradiol production. However, it decreases progesterone synthesis and the expression of different proteins involved in steroidogenesis [3β-HSD, cytochrome P450 (CYP11A1), steroidogenic acute regulatory protein (StAR)] (Tosca et al., 2006b). Metformin reduces follicle-stimulating hormone (FSH) but not forskolin-stimulated aromatase expression and activity in an AMP-activated protein kinase-independent manner in a human granulosa cell line (Rice et al., 2013). Also in human granulosa cells, metformin decreases androgen synthesis, by directly inhibiting cytochrome P450 17alpha-hydroxylase (Cyp17) activity (La Marca et al., 2000). In contrast, it has been shown that AMPK could improve androgen production by adrenal cells (Hirsch et al., 2012). Even if no studies have shown a role of AMPK in ovarian steroidogenesis *in vivo*, this has been largely demonstrated *in vitro*.

A total α1AMPK knock out mouse model has been developed (Tartarin et al., 2012a). The male α1AMPK^−∕−^ have high levels of testosterone that are not due to adrenal disorders or to glucorticoid resistance but to hyperactive Leydig cells (Tartarin et al., 2012a). Indeed, the Leydig cells of these animals have an increased volume, an altered endoplasmic reticulum area, a high intratesticular cholesterol concentration and a greater expression of proteins involved in steroid production (Tartarin et al., 2012a). These data accord with those described previously *in vitro* in granulosa cells but also in Leydig cells in response to a modulation of AMPK activity either pharmacologically or genetically. Certainly in MA-10 and MLTC-1 Leydig cells, AMPK activation inhibits cAMP-induced steroidogenesis by repressing the expression of key regulators of steroidogenesis, including the cholesterol carrier, StAR and the nuclear receptor Nr4a1 (Abdou et al., 2014). In the latter study, the authors suggest that some AMPK-sensitive element(s) containing sites for nuclear receptors of NR4A1 are located in the StAR promoter and are required for elevated cAMP dependent activation (Abdou et al., 2014). This suggests that activation of AMPK reduce the activity of NR4A1 and StAR expression. Furthermore, in primary rat Leydig cells, resveratrol, an AMPK agonist impairs human chorionic gonadotropin (hCG)-mediated testosterone production by repressing StAR expression (Svechnikov et al., 2009). In humans, the association of increased steroid production and the inhibition of AMPK could be associated to the Peutz-Jeghers Syndrome (PJS) (Ham et al., 2013). Peutz-Jeghers syndrome is an autosomal-dominant disorder that arises as a consequence of mutations in the serine/threonine kinase 11 (STK11) gene that encodes LKB1. In these PJS patients, excess estrogen and an increase in testicular aromatase expression is associated with a decrease in AMPK phosphorylation in the testis (Ham et al., 2013). Thus, AMPK could be a molecular modulator that inhibits gonadal steroidogenesis to preserve cellular energy homeostasis and prevent excess steroid production.

An important study for human health found that when human and mouse fetal testes were cultured in the presence of metformin, there was a reduction in testosterone secretion and mRNA of key factors which are involved in steroidogenesis (Tartarin et al., 2012b). This was also associated with an increase in lactate production. Furthermore, *in vivo* administration of metformin during pregnancy reduced the testicular size of fetal and neonatal testes. Although the number of germ cells was not altered by metformin treatment, the number of Sertoli cells was reduced in both fetal and neonatal testes. Interestingly the androgen producing Leydig cell population was only reduced in the fetal period at 16 days post-coitum (Tartarin et al., 2012b). This study presented a potentially harmful effect of metformin treatment on the development of fetal testes (Tartarin et al., 2012b). These effects were likely to result from a metformin-stimulated AMPK-mediated reduction in cellular proliferation (Kayampilly and Menon, 2012), indicating that the reduction in steroidogenesis occurred as a result of reduced testicular growth.

## Proliferation and survival of somatic gonadal cells

Gonadal somatic cells comprise the granulosa, cumulus and theca cells of the ovary, and the Sertoli and Leydig cells of the testis. Proliferation and survival of somatic cells are indispensable for fertility. Indeed, it is well known that proliferating granulosa cells support the progression of follicular growth and oocyte maturation. In males, testis size and sperm production are directly correlated to the total number of adult Sertoli cells. Regulation of proliferation and survival processes involves different hormones including FSH. As AMPK has previously been described as inhibiting proliferation of somatic cells (Tosca et al., 2010; Hardie, 2011; Kayampilly and Menon, 2012; Riera et al., 2012), we will examine the proliferative role of AMPK for these critical cell types.

In *C. elegans*, AMPK promotes survival and arrests germline development during nutrient stress (Fukuyama et al., 2012). More precisely, AMPKα1 and AMPKα2 (aak-1 and aak-2), the two catalytic α subunits of AMP-activated protein kinase, regulate germline quiescence by suppressing activity of target of rapamycin complex 1 (TORC1) that is involved in cell growth, cell proliferation, cell motility, cell survival, protein synthesis, and transcription. Similarly in rat Sertoli cells, Riera et al. (2012) observed that activation of AMPK induces a decrease in FSH-stimulated Sertoli cell proliferation through a phosphatidylinositol 3-kinase (PI3K)/protein kinase B (Akt)/mTORC1 mechanisms but also an increase in cyclin dependent kinase inhibitor (CDKI, p19INK4d, p21Cip1, and p27Kip1) expression (Riera et al., 2012). In agreement with these data, Tosca et al. (2010) observed that metformin-induced AMPK activation reduces cell growth, protein synthesis and MAPK ERK1/2 and ribosomal protein S6 kinase (p90rsk) phosphorylation in response to insulin-like growth factor 1 (IGF1) in cultured bovine granulosa cells. Furthermore, Kayampilly and Menon observed that exposure of rat granulosa cells with a pharmacological activator of AMPK increased p27kip expression, an inhibitor of the cell cycle (Kayampilly and Menon, 2009). These latter authors have also observed that activation of AMPK induced by dihydrotestosterone (DHT) treatment decreases granulosa cell mitogenesis and consequently could contribute to ovulatory dysfunction observed in hyperandrogenic states (Kayampilly and Menon, 2012).

## Maturation of germ cells

### The oocyte

The role of AMPK in mammalian oocyte maturation is strikingly species specific. AMPK improves resumption of oocyte meiosis in mice (Chen et al., 2006; Downs and Chen, 2006; Larosa and Downs, 2007; Chen and Downs, 2008) but not in rats (Downs, 2011) and pharmacological activation of AMPK blocks nuclear oocyte maturation in pigs and cattle (Mayes et al., 2007; Tosca et al., 2007b; Santiquet et al., 2014). The oocyte is reliant on the metabolism of lactate and pyruvate from the tricarboxylic acid cycle (TCA cycle) and oxidative phosphorylation for most of its energy stores (Biggers et al., 1967; Leese and Barton, 1984; Roberts et al., 2002). In human granulosa cells, AMPK could be also involved in lactate production which is important for follicular development (Richardson et al., 2009). The cumulus-oocyte complex (COC) also metabolizes glucose via numerous important pathways such as the pentose phosphate pathway and glycolysis. These metabolic pathways are critical for successful oocyte maturation and resumption of meiosis (Downs and Mastropolo, 1994; Downs et al., 1996; Sutton et al., 2003a,b). Cyclic adenosine monophosphate (cAMP), synthesized by adenylate cyclase downstream of the pentose phosphate pathway, is an important negative regulator of meiotic maturation. It is well known that degradation of cAMP by phosphodiestrases triggers resumption of meiosis. The depletion of cAMP results in an increase in the AMP/ATP ratio. As AMP levels rise, AMPK is triggered and activates a number of enzymes involved in energy producing pathways and inhibiting energy consuming pathways (Downs et al., 2002). This makes AMPK critically important for oocyte developmental competence.

In mouse oocytes, the α1AMPK subunit is more abundant than α2AMPK. Immunolocalization of the α1 catalytic subunit of AMPK showed an association with condensed chromatin and the meiotic spindle but not in the spindle poles or midbody. In the absence of α1AMPK specifically in the oocyte, a decrease in mdm2 protein level, a strong negative regulator of p53, leads to an increase in the p53 content and probably induces cell cycle arrest as shown by the few number of oocyte fertilized in IVF or by the lower litter size in comparison to control mice (Bertoldo et al., 2014b, 2015). AMPK activation increases the rate of germinal vesicle breakdown (GVBD), spindle formation and polar body (PB) extrusion whereas the kinase has no effect on peripheral movement of the spindle. The meiosis-inducing actions and localization of AMPK are regulated by microtubule spindle integrity during mouse oocyte maturation (Ya and Downs, 2014). Interestingly, in mice, fatty acid oxidation is required for AMPK-induced maturation *in vitro* (Vasangkar and Downs, 2013).

The AMPK activator, AICAR, is a potent stimulator of maturation in mouse cumulus cell-enclosed oocytes (CEO) and denuded oocytes (DO), but only marginally stimulatory in rat CEO and ineffective in rat DO (Downs, 2011). AICAR and compound C produced contrasting results on polar body formation in cultured CEO in rat and mouse. Active AMPK was colocalized with chromatin after GVBD in rat and mouse oocytes, but did not appear at the spindle poles in rat oocytes as it did in mouse oocytes. These data highlight significant differences in meiotic regulation between the two species (Downs, 2011).

Contrary to results obtained with mouse oocytes, bovine and porcine oocyte meiosis is inhibited by activators of AMPK which is activated by AMP, the degradation product of cAMP (Bilodeau-Goeseels et al., 2007; Mayes et al., 2007; Bilodeau-Goeseels, 2011). During oocyte *in vitro* maturation (IVM), Santiquet et al. (2014) cultured porcine oocytes in the presence of AICAR and assessed the oocytes response in reference to oocyte nuclear maturation and cumulus cell expansion. Nuclear maturation was inhibited, however, this effect was only observed in cumulus enclosed oocytes, suggesting that cumulus cells are essential for AICAR's effect on oocyte maturation. In addition, AICAR inhibited cumulus expansion, which normally occurs in response to FSH and/or epidermal growth factor (EGF) during IVM (Harper and Brackett, 1993; Lonergan et al., 1996; Ritter et al., 2015). The results in porcine are supported by those in bovine where supplementation of metformin during embryo *in vitro* production resulted in AMPK mediated activation of TSC2 (Pikiou et al., 2015) and probably a reduction in TOR complex signaling and protein synthesis inhibition. In bovine COCs, metformin blocks meiotic progression at the germinal vesicle stage, activates AMPK, and inhibits MAPK3/1 phosphorylation in both the oocytes and cumulus cells during *in vitro* maturation. Moreover, cumulus cells were essential for the effects of metformin on bovine oocyte maturation, whereas MAPK ERK1/2 phosphorylation was not (Tosca et al., 2007b). While the precise targets of AMPK in the COCs are not entirely known, AMPK has been shown to modulate protein synthesis in various cell types (Hormon et al., 2002; Proud, 2004) and proteins involved in communication with somatic cells (see next section).

As observed in the large animal species, AMPK signaling keeps nemertean oocytes from maturing (Stricker et al., 2013). Unlike in mice, where the onset of oocyte maturation (germinal vesicle breakdown, GVBD) is blocked by cAMP and triggered by AMPK, oocytes of the marine nemertean worm Cerebratulus undergo GVBD in response to cAMP elevations and AMPK deactivation (Stricker, 2011). In addition these effects are observed only in the absence of the surrounding somatic cells (Stricker et al., 2010). These results also provide evidence for a novel GVBD-regulating mechanism involving AMPK deactivation by cAMP-mediated S485/491 phosphorylation and further highlight the highly species specific effects of AMPK in regard to oocyte maturation.

### Male germ cells

αAMPK is present in male germ cells of oyster (Guévelou et al., 2013), chicken (Nguyen et al., 2014) and mammals (Hurtado de Llera et al., 2012a; Tartarin et al., 2012a; Cordova et al., 2014). In oyster, it is more highly expressed in male gonad than in female and its expression is more important during the first stage of gametogenesis when germ cells proliferate (Guévelou et al., 2013).

To traverse the female reproductive tract, it is essential that mammalian spermatozoa acquire the functional competence to achieve this objective in order to successfully fertilize the oocyte. These functional markers include motility, capacitation, hyperactivation and the acrosome reaction (Hurtado de Llera et al., 2015). It was recently demonstrated that AMPK protein is highly expressed in ejaculated boar and chicken spermatozoa (Hurtado de Llera et al., 2012a; Nguyen et al., 2014), and in mouse epididymal sperm (Tartarin et al., 2012a; Bertoldo et al., 2014a) and that it localizes in the head of the spermatozoon and in the midpiece of the flagellum (Hurtado de Llera et al., 2013). In the boar, pharmacological inhibition of AMPK lead to a reduction in motility (Hurtado de Llera et al., 2012a) while concomitantly causing changes in mitochondrial membrane potential, sperm plasma membrane fluidity and organization and acrosome integrity (Hurtado de Llera et al., 2013; Martin-Hidago et al., 2013). Similar results have been described in the chicken. These studies highlight the conservation of the AMPK function in sperm activity.

Interestingly, Hurtado de Llera *et al*. noted that the majority of studies to date that had studied sperm physiology as a function of AMPK had only done so under conditions where AMPK was inhibited (Hurtado de Llera et al., 2012b, 2015) or was genetically silenced (Tartarin et al., 2012a). Consequently they conducted a study to assess sperm physiology while activating AMPK indirectly. They observed under extended periods (24 h) of AMPK activation, boar spermatozoa had reduced motility, acrosome membrane integrity and organization and fluidity of the plasma membrane which was associated with an increase in lipid disorganization (Hurtado de Llera et al., 2015). As these processes are critical under the different environmental conditions experienced by spermatozoa when transiting through the female reproductive tract to accomplish fertilization, it becomes obvious from studies carried out so far that an optimal level of AMPK activation is essential for regulating spermatozoa function (Hurtado de Llera et al., 2015).

Nakada *et al*. demonstrated that spermatogenesis is intimately linked to mitochondrial respiration (Nakada et al., 2006), and recently Pellicione et al. associated asthenozoospermia with abnormal mitochondrial ultrastructure (Pelliccione et al., 2011). Therefore, it is likely that the motility disturbances observed in our α1AMPK KO model and in the LKB1 KO model that presented with abnormalities in spermatozoa functionality and morphology (motility and head morphology) (Towler et al., 2008; Tartarin et al., 2012a), was directly linked to mitochondrial dysgenesis. Similarly, incubation of sperm from boar with an AMPK inhibitor, compound C, lead to a reduction in motility (Martin-Hidago et al., 2013). Surprisingly, a mouse model inactivated for an oxidative stress sensor protein like glutathione peroxydase 4 (Liang et al., 2009; Schneider et al., 2009) is described with structural abnormalities in spermatozoa analogous to those observed in α1AMPK KO, suggesting that mitochondria dysfunction could affect oxidative stress oxidative status.

Diabetic and infertile men present with a decrease in anti-oxidant concentrations and an increase in ROS generation in their semen, even before cryopreservation, demonstrating that the sperm from this group are at increased risk of oxidative damage (Lewis et al., 1995; Garcez et al., 2010). Several studies have demonstrated that metformin can reduce the levels of oxidative DNA damage and afford anti-oxidant protection (Attia et al., 2009; Onken and Driscoll, 2010; Martin-Montalvo et al., 2013). Metformin has been shown to guard against diabetes-induced genomic instability in sperm cells and the bone marrow of diabetic rats (Attia et al., 2009), so we can hypothesize that the use of metformin in diabetic patients would have no negative effect in the integrity of spermatozoa. However, we cannot exclude consequences on the paternal genome for oocyte fertilization. For example, treatment of murine sperm with high concentration of metformin increased histone deacetylase activity (Bertoldo et al., 2014a). Curiously, treatment of sperm with known natural activators of AMPK such as resveratrol or a synthetic activator like metformin, present positive effects such as reduction in DNA damage and lipid peroxidation (Bertoldo et al., 2014a). A study in the wood frog (*Rana sylvatica*), a species with a high freeze tolerance, has revealed that AMPK was more activated in liver and muscle tissue, thus presenting AMPK as a molecule with cryoprotective properties (Rider et al., 2006). Subsequently modification of AMPK has been exploited in the freezing protocols of mammalian semen (Bertoldo et al., 2014a; Cordova et al., 2014). Metformin was used in mouse semen extender (Bertoldo et al., 2014a) and following thawing, spermatozoa showed an improvement in fertilization capability *in vitro*. This was associated with a reduction in the number of abnormal zygotes following IVF when mouse spermatozoa was frozen in the presence of metformin compared to controls (Bertoldo et al., 2014a). AMPK was also modulated in stallion semen extender where there was an improvement in sperm quality post-thaw (Cordova et al., 2014). As the reports of AMPK presence in spermatozoa are growing in number, we believe it is reasonable to assume that AMPK activity is likely required for optimal mammalian spermatozoa physiology.

## Germinal and somatic cells interactions

### Cumulus-oocyte complex

Gap junction communication between cumulus cells and oocytes is crucial for oocyte meiotic maturation and to acquire full developmental competence (Gilchrist et al., 2004; Gilchrist, 2011; Li and Albertini, 2013). Such that maintenance of gap junction communication and delayed meiotic resumption have been shown to increase oocyte developmental competence (Thomas et al., 2004; Gharibi et al., 2013). Gap junction communication between the oocyte and the surrounding cumulus cells is established by the formation of bidirectional channels. The connexins family, which compose gap junctions is involved in oocyte/cumulus cell communication and allows passage of ions and small organic molecules. Loss of connexin 37 and connexin 43 in mouse oocytes or cumulus cells impaired fertility through inhibiting oocyte growth and folliculogenesis (Winterhager and Kidder, 2015). Electron microscopic analysis has shown that junctions between granulosa cells and oocytes are altered or absent as in connexin 37^−∕−^ mice (Simon et al., 1997). In the mouse, deletion of α1AMPK specifically in the oocyte lead to a reduction in connexin 37 between the oocyte and cumulus cells at the Metaphase II stage which was associated with reduced fertility following IVF, and suggests a reduction in gap junction communication (Bertoldo et al., 2015). Reductions in connexin 26 and connexin 37 expression were also described in a diabetic mouse model, where oocyte quality is poor (Ratchford et al., 2008). In an non-mammalian example, Alesutan et al., demonstrated that active AMPK decreased connexin 26 abundance in the cell membrane in xenopus oocytes (Alesutan et al., 2011), suggesting disparate regulation of gap junction communication by AMPK between species.

Deletion of α1AMPK in oocytes leads to reductions in other proteins associated with intercellular communication within the cumulus oocyte complexes (Bertoldo et al., 2015). These include N-cadeherin and β-catenin (markers for adherens junctions) and occludin (a marker for tight junctions) (Bertoldo et al., 2015). The cumulus-oocyte complex interacts with granulosa cells through adhesion junctions composed of proteins such as E-cadherin and N-cadherin (Rufas et al., 2000; Machell and Farookhi, 2003). Expression of N-cadherin for example, increases throughout maturation, fertilization and early embryogenesis (Ziv et al., 2002), and N-cadherin mediated cell contact is associated with the maintenance of meiotic arrest (Peluso, 2006). Deregulation of these proteins impacts oocyte maturation, fertilization and early embryogenesis (Ziv et al., 2002; Peluso, 2006).

Furthermore in the bovine, it was recently demonstrated that the transzonal processes (TZP) that traverse the zona pellucida transfer RNA molecules from cumulus cells to the oocyte (Macaulay et al., 2014). It was proposed that these TZPs are held in place by adherens like junctions (Macaulay et al., 2014) and are critical for oocyte developmental competence. During repair of lung capillary endothelium α1AMPK promotes the development of intercellular adherens junctions by binding with N-cadherin and contributes to repair (Creighton et al., 2011). This supports the notion that AMPK may have a critical role in oocyte developmental competence by maintaining open oocyte-somatic cell communication channels through at least gap, adherens and tight junctions. Taken together the literature supports the concept that AMPK plays a crucial role in maintaining metabolic and molecular intercellular coupling between the oocyte and its somatic cells and breakdown of this coupling results in reduced oocyte developmental competence.

### Blood testis barrier

As in the cumulus oocyte complex, male germ cells are closely linked their support cells; the Sertoli cells during their maturation. Sertoli cells have an important role in the shaping of the spermatid head for example (Kierszenbaum and Tres, 2004). Different transgenic mouse models show that AMPK plays a role in intra-testicular communication. Absence of the upstream AMPK kinase, LKB1 reduced mature spermatozoa production associated with abnormal acrosome morphology and a defect in Sertoli cell polarity and testicular junctional complexes (Towler et al., 2008). Patients with Peutz-Jeghers syndrome present a similar phenotype with alteration of sperm production associated with modifications of tight junctions in the blood-testis-barrier (Ulbright et al., 2007; Chen et al., 2012; Tanwar et al., 2012). The disruption of the α*1AMPK* gene in the whole murine testis induced altered sperm morphology without presenting abnormalities in Sertoli cell nucleus polarization (Tartarin et al., 2012a). Nonetheless, transmission electron microscopy analyses have shown the presence of some disrupted Sertoli cell/elongated spermatid germ cell junctions (Tartarin et al., 2012a). Interestingly, a similar phenotype in sperm head or midpiece morphology has been already described in mice deleted for adhesion molecules like nectin-2 (Mueller et al., 2003) or Tslc1 (Surace et al., 2006). The fact that absence of the α*1AMPK* gene lead to a mild phenotype in contrast to LKB1 could be explained by the activation of LKB1 through several other AMPK-related kinases present in the testis such as microtubule-associated protein/microtubule affinity-regulating kinases (MARK2) (Bessone et al., 1999) or SNRK (Jaleel et al., 2005) as a compensatory mechanism. This hypothesis is supported by the decrease in phosphorylation of MARK in testis in LKB1-KO mice (Kojima et al., 2007; Tanwar et al., 2012). The reduction and/or incorrect relocalization of markers of adherens junctions (β-catenin and N-Cadherin) (Kopera et al., 2010) and tight junctions (occludin and ZO-1) (Kopera et al., 2010) in Sertoli cells from LKB1-KO mice suggests a loss of contact with germ cells leading probably to alteration in germ cell shape (Kleymenova et al., 2005). Notably, altered β-catenin expression has previously been described to compromise Sertoli cell function and the maturation of germ cells and lead to infertility (Lee et al., 2005; Tanwar et al., 2010; Kerr et al., 2013).

The use of the AMPK activator (AICAR) can also influence junction complex integrity in rat Sertoli cells as has been described by Galardo et al. (2010). Rat Sertoli cells incubated with AICAR stabilized ZO-1 protein as observed by immunofluorescence (Galardo et al., 2010). The use of EGTA to limit the free calcium concentration in culture medium induced a redistribution of ZO-1 between Sertoli cells. Addition of AICAR or adenosine, in the presence of EGTA permitted the rescue of ZO-1 distribution to normal conditions at the cell membranes (Galardo et al., 2010). As in oocytes, the cAMP/PKA pathway is modified in the absence of α1AMPK in Sertoli cells, raising the question about the interaction between AMPK/cAMP signaling and the functionality of the blood-testis barrier permeability. Indeed, the cAMP signal has been described to be involved for the formation and maturation of male germ cells (Scobey et al., 2001), and can perturb junctions in rat Sertoli cells (Lui and Lee, 2005).

## Interface between AMPK and modification of metabolism

Diet restriction is well known to promote longevity and reduce fertility in several species like *C. elegans, drosophila melanogaster*, birds and mammals. Diet restriction induces a negative energy balance which activates some energy sensors such as AMPK and the sirtuins which promote respiration and energy production by mitochondria. In mice, a deficiency in LKB1 or AMPK in mature Sertoli cells negatively impacts mitochondrial function, and has been associated with loss of quiescence and an activation of cell proliferation (Bertoldo et al., 2013). The association between nutrient availability, mitochondrial function and fertility has been already observed in invertebrates. In *C. elegans* the germinal stem cells regulate longevity through the TOR pathway (Arrantes-Oliveira et al., 2002), and similarly in drosophila TOR signaling is involved in the regulation of female germinal stem cell proliferation as a function of the availability of nutrients (Drummond-Barbosa and Spradling, 2001; Lafever and Drummond-Barbosa, 2005; Lafever et al., 2010; Shyh-Chang et al., 2013). These results are also observed in Sertoli cells where stimulation with an AMPK activator such as metformin or AICAR has consequences on lactate production and the increase in glucose transport (Galardo et al., 2007). One hypothesis of the action of metformin, is an indirect effect: an inhibition of the respiratory chain in mitochondria leading to an increase in lactate production, and in the AMP: ATP ratio inducing the activation of AMPK. On the other hand, the inactivation of α1AMPK in Sertoli cells, reduces the expression of mitochondrial markers (cytochrome c and PGC1a) and the ATP content, and increases the lactate production (Bertoldo et al., 2013). The increase in lactate, in this case, could be due to a switch in the cell between energy production by respiration to the aerobic glycolysis. Thus, α1AMPK deficiency enhances the Warburg Effect which can be associated with increased cell proliferation *in vitro* (Faubert et al., 2013). In addition, glycolysis could increase the allocation of glucose carbon into lipids and explain the increase in lipid vesicles. We cannot exclude that a modification in lipid metabolism in Sertoli cells has a consequence on germ cells. Indeed, some studies have described lipid transport from the Sertoli cells to the germ cells (Saether et al., 2003). Moreover, several recent studies using mice deficient in genes related to lipid metabolism, have described that the accumulation of excess lipid droplets in Sertoli cells resulted in impaired spermatogenesis (Coussens et al., 2008). Therefore, a balance of lipid metabolism in Sertoli cells is essential for normal spermatogenesis (Selva et al., 2004).

Ratchford et al. have hypothesized that abnormalities in oocyte metabolism, such as that observed in diabetes, could potentially preprogramme the oocyte for unfavorable outcomes after fertilization (Ratchford et al., 2007). Furthermore, Wang et al. (2009) concluded that maternal diabetes results in numerous oocyte deficiencies. Glucose metabolism is essential for successful oocyte maturation and the recommencement of meiosis (Downs and Mastropolo, 1994). It is well known that mitochondria can influence the developmental competence of the oocyte (Thouas et al., 2004). Certainly mitochondria play a key role in cellular energy generation, the control of cell death (Perez et al., 2000) and the dynamic process of meiosis including DNA reorganization (Wang et al., 2009). In the case of diabetes, mitochondria are abnormally distributed around the spindle or in the oocyte cytoplasm (Wang et al., 2009). Ratchford et al. observed that under hyperglycaemic conditions, phosphorylated ACC, a downstream target of AMPK and phosphorylated AMPK were both decreased in diabetic oocytes, demonstrating decreased AMPK activity (Ratchford et al., 2007). Diabetic oocytes were also metabolically perturbed leading to altered AMPK activity. Interestingly, increasing AMPK with AICAR in these oocytes during the preovulatory phase corrected the metabolic and meiotic perturbations observed. For these crucial activities in oocyte maturation, mitochondrial redistribution, activity or dysfunction have been suggested as markers of oocyte quality and are strongly related to fertilization rates and embryo development (Van Blerkom, 2004; Wang et al., 2009).

During the last decade a variety of natural ligands and synthetic ligands have been shown to activate AMPK including resveratrol (Baur et al., 2006), sulforaphane (Choi et al., 2014), niacin (Thirunavukkarasu et al., 2006), berberine (Brusg et al., 2006), metformin (Zhou et al., 2001), and thiazolidinediones (Fryer et al., 2002). Some of these compounds have non-linear dose-response characteristics, such as that of hormesis and have the ability to inhibit the mitochondrial complex I at elevated concentrations that mimick diet restriction (Gems and Partridge, 2008). The hypothesis of hormesis lends weight to differences in phenotype associated with differences of metformin concentration. High metformin concentrations (approximately 5 mM) is enough to inhibit the respiratory chain complex 1 in mitochondria leading to an increase in the AMP/ATP ratio (El-Mir et al., 2000; Owen et al., 2000) and different metformin concentrations induce increases in oxidant defenses as well as an extension of lifespan (Onken and Driscoll, 2010; Martin-Montalvo et al., 2013). The difference in species sensitivity has been already observed as mouse tissue is 10 fold less sensitive than human tissue (Tartarin et al., 2012b).

Metformin is a good example for mimicking diet restriction, because in mouse liver, metformin has been shown to induce a similar transcription pattern to diet restriction especially (Dhahbi et al., 2005). However, similar effects have been described in *C. elegans* where metformin administration increases the lifespan and produces several diet restriction-like phenotypes such as reduction in fecundity and a decrease in fat storage in animals which are fed *ad-libitum* (Onken and Driscoll, 2010). In drosophila, metformin exposure for 7 days at 25 and 50 mM concentration increases significantly the number of eggs laid in contrast to untreated controls. But after 14 days of treatment, egg-laying in females on 25 mM metformin was similar to controls and at 50 mM of metformin the females laid significantly fewer eggs (Onken and Driscoll, 2010). Interestingly in flies, metformin targets AMPK and inhibits the TOR pathway (Kalender et al., 2010; Slack et al., 2012). From the reports to date, we can conclude that effects on fertility (increases or reductions in the number of egg laid depending the time and concentration of metformin treatment) remains partially understood and controversial (He and Wondisford, 2015).

SIRT1 is widely regarded as a critical regulator of energy homeostasis and is implicated in a wide variety of cellular processes including metabolic diseases, cancer, aging, and reproduction (Bordone and Guarente, 2005; Brooks and Gu, 2009; Haigis and Sinclair, 2010). Furthermore it is known to interact with AMPK (Fulco et al., 2008; Narala et al., 2008; Canto and Auwerx, 2009). We have recently provided evidence in the oocyte that α1AMPK could be involved in chromatin remodeling, because we observed an increase in acetylation of H3 histone in oocytes from α1AMPK knockout oocytes (Bertoldo et al., 2015). This was correlated, as expected with a reduction in histone deacetylase SIRT1 expression *in vivo*. *In vitro* Sirt1 has the ability to deacetylate histone substrates in a NAD^+^-dependent manner (Vaquero et al., 2004b) and hyperacetylation occurs when SIRT1 is knocked down (Vaquero et al., 2004b). Male mice deficient in SIRT1 present with altered germ cell maturation and increased DNA damage in germ cells (Coussens et al., 2008). Together, these data suggest that AMPK can modify oocyte proteins and histone acetylation status. These observations could be linked to other reports such as those relating to the aorta and heart tissue where a decrease in AMPK and SIRT1 expression is associated with increased H3 acetylation (Bendale et al., 2013). Interestingly, acetylation of histones H3 and H4 appear to be linked to an overexpression of connexin 43 in a prostate cell line (Ogawa et al., 2005; Hernandez et al., 2006), and PGC1α and p53 can modify their accessibility (Vaquero et al., 2004a; Wakeling et al., 2009; Nelson et al., 2012), possibly suggesting some level of control of intercellular communication and apoptosis. Interestingly, inadequate histone deacetylation causes changes in gene expression, which can lead to embryopathy in mice (Akiyama et al., 2006).

## Conclusion

The involvement of AMPK in fertility control is conserved throughout several animal species from the oyster, *C. elegans*, drosophila, birds and mammals. Its expression is present in different compartments of the ovary and testis and through the different stages of maturation of germ cells, germline stem cells to oocytes and spermatozoa. Apart from its classical functions on metabolism, proliferation and anti-inflammatory effects observed in the gonad, AMPK is also able to modulate steroidogenesis, and to impact morphology and normal nuclear maturation of germ cells though interaction of germ cells with their nurse somatic cells. Some mechanisms elucidated are directly linked with mitochondrial function and junctional proteins. Despite the possibility of different sub-unit combinations of AMPK, absence of only α1AMPK leads to moderate failure of fertility in both sexes.

## Funding

This review was financially supported by ANR “Fertinergy grant.”

### Conflict of interest statement

The authors declare that the research was conducted in the absence of any commercial or financial relationships that could be construed as a potential conflict of interest.
